# Development of Bioresponsive Poloxamer-Based Self-Nanoemulsifying System for Enhanced Febuxostat Bioavailability: Solidification Strategy Using I-Optimal Approach

**DOI:** 10.3390/pharmaceutics17080975

**Published:** 2025-07-28

**Authors:** Abdelrahman Y. Sherif, Ehab M. Elzayat

**Affiliations:** Department of Pharmaceutics, College of Pharmacy, King Saud University, P.O. Box 2457, Riyadh 11451, Saudi Arabia; ashreef@ksu.edu.sa

**Keywords:** poloxamer, propylene glycol, SNEDDS, febuxostat, solidification

## Abstract

**Background/Objectives:** The major limitations of self-nanoemulsifying systems include complex processing and expensive instrumentation required for solidification approaches. In this study, smart poloxamer-based solidification strategies were used to develop and optimize febuxostat-loaded formulations. **Methods:** A self-nanoemulsifying drug delivery system (SNEDDS) component was selected based on solubility and emulsification tests. The influence of poloxamer molecular weight (low or high) and its concentration (2–10% *w*/*w*) on formulation performance was assessed through the design of experiments. Finally, in-vitro melting assessment and a comparative dissolution test were performed on the optimized SNEDDS formulation. **Results:** Imwitor 988 and Tween 20 were selected to prepare the formulations. Increasing the molecular weight and concentration of the poloxamer significantly increased the temperature and time required for the melting of the SNEDDS formulation. The optimized SNEDDS formulation comprised 3.98% *w*/*w* poloxamer 188, which melts at 36 °C within 111 s. In-vitro melting showed that the formulation completely converted to a liquid state upon exposure to body temperature. Finally, the optimized SNEDDS formulation exhibited superior dissolution efficiency (96.66 ± 0.28%) compared to raw febuxostat (72.09 ± 4.33%) and marketed tablets (82.23 ± 3.10%). **Conclusions:** The poloxamer-based approach successfully addressed the limitations associated with conventional solidification while maintaining superior dissolution performance. Therefore, it emerges as a promising alternative approach for enhancing the bioavailability of poorly water-soluble drugs.

## 1. Introduction

Oral administration is the predominant therapeutic route for all forms of pharmaceutical dosage owing to its inherent patient acceptability, simplicity of administration, and relative cost-effectiveness [1]. However, this conventional approach encounters substantial obstacles in the formulation of lipophilic therapeutic molecules [2]. The poor solubility and slow dissolution velocity of these molecules within the hydrophilic components of the gastrointestinal tract substantially influence their bioavailability [3].

Contemporary innovations in drug delivery have gravitated toward lipid-based formulations capable of counteracting solubility, which affects the bioavailability of therapeutic molecules [4]. Specifically, self-nanoemulsifying drug delivery systems (SNEDDS) have received extraordinary attention because of their spontaneous nanoemulsion genesis within the gastrointestinal aqueous environment. The thermodynamic stability of anhydrous formulations and their ability to spontaneously transform into oil-in-water emulsions enable drug solubilization [5]. This improves oral bioavailability by enhancing the concentration gradient driving force and promoting lymphatic uptake, thereby reducing exposure to hepatic first-pass metabolism [6].

The aforementioned characteristics of self-emulsifying formulations make them effective in improving the bioavailability of lipophilic febuxostat, which exhibits an absolute bioavailability of 49% [7]. Consequently, pharmaceutical researchers have been motivated to develop febuxostat-loaded self-emulsifying systems to overcome oral bioavailability barriers [8,9,10]. Moreover, the prepared formulations are solidified using adsorption, extrusion/spheronization, freeze-drying, and film formation approaches to overcome the limitations of formulation leakage associated with liquid SNEDDS [11]. However, the approaches used to solidify SNEDDS require additional steps and expensive instrumentation, which increases the production time and cost of the final product. This requires a different approach to address the limitations of previously established solidification approaches.

Thermoresponsive polymers provide an innovative alternative solidification strategy based on physicochemical transitions triggered by physiological temperature changes [12]. Among them, poloxamers are composed of triblock copolymers (PEO-PPO-PEO) that undergo changes in the arrangement of polymeric molecules from discrete polymer chains to micellar structures when exposed to high temperatures [13]. On the other hand, upon cooling, their polymeric molecules arrange as discrete polymer chains. Therefore, the incorporation of a crosslinking agent (propylene glycol) into the formulation allows its solidification by forming a hydrogen bond between the hydroxyl groups of poloxamer and propylene glycol [14,15]. However, the transition temperature at which this arrangement occurs depends on the type and concentration of poloxamer [16]. Therefore, optimization using the design of experiments (DoE) approach overcomes the limitations of time consumption and lack of accuracy when compared to traditional method [17]. Moreover, the DoE approach also provides a clear illustration of the complex interactions between the studied factors and their effects on critical formulation attributes [18,19].

In this study, we aimed to develop and optimize a solid SNEDDS for febuxostat using a systematic DoE approach. Using this approach, we evaluated the influence of critical formulation variables on the performance of the prepared formulation and selected the optimized SNEDDS formulation. Additionally, we evaluated the in-vitro dissolution performance of raw febuxostat, marketed tablets, and the optimized SNEDDS to investigate the impact of the proposed approach on formulation performance.

## 2. Materials and Methods

### 2.1. Materials

Febuxostat was donated by SPIMACO (Buraydah, Qassim, Saudi Arabia). Oils were obtained from different sources—oleic acid was obtained from Avonchem (Cheshire, UK), Imwitor-988 from Sasol Germany GmbH (Hamburg, Germany), peceol from Gattefosse (Saint-Priest, France), Captex 355 EP/NF from Abitec Corporation (Janesville, WI, USA), and soybean oil from John L. Seaton & Co., Ltd., Croda International Plc. (Goole, East Yorkshire, UK). Surfactants used in this study included Tween-20 (BDH, Poole, UK), Tween-80 (Loba Chemie, Mumbai, India), Labrasol (Gattefosse, Saint-Priest, France), Kolliphor-EL (BASF, Ludwigshafen, Germany), and HCO-30 (Nicole Chemical Co., Tokyo, Japan). Cosurfactant propylene glycol was obtained from Winlab Laboratory (Market Harborough, Leicestershire, UK).

### 2.2. Drug Quantification

A Dionex ultraperformance liquid chromatography (UPLC) system (Thermo Scientific, Bedford, MA, USA) was used to quantify the drug concentrations within the analyzed samples. The mobile phase, consisting of an aqueous 0.1% formic acid in acetonitrile (30:70 *v*/*v*), was eluted through a BEH C18 column (2.1 × 50 mm, 1.7 μm) at a flow rate of 0.4 mL/min. The temperature of the used column was maintained at 25 ± 0.1 °C using a Dionex column oven chamber. The absorbance of the febuxostat was measured at the maximum wavelength (318 nm) using a Dionex Photodiode Array detector.

### 2.3. Solubility of Febuxostat

The solubility of febuxostat was tested to select the optimum oil phase and surfactant for preparing SNEDDS formulations. An excess amount of febuxostat was mixed with the tested agent (oil or SNEDDS formulation) in a 5 mL glass beaker for one day at 1000 rpm. The mixture was then centrifuged at 10,000 rpm for 30 min to settle the undissolved drug, and drug solubility was tested using the developed UPLC method. The clear supernatant was collected using a pipette and transferred to a 2 mL Eppendorf tube placed on a balance for precise weighing. Subsequently, 1.8 mL of an organic solvent (acetonitrile) was added to extract the drug, and the sample was subjected to an appropriate dilution for drug quantification.

### 2.4. Emulsification Study

This study was performed to determine the emulsification efficiency of the surfactants and oils. Each surfactant (Tween 20, Tween 80, Kolliphor EL, labrasol, and HCO-30) was individually mixed with Imwitor 988 oil and propylene glycol cosurfactant and then incubated for at least two hours at 40 °C to facilitate miscibility between components. The prepared mixture (surfactant, Imwitor 988, and propylene glycol) was diluted with Milli-Q water (1:1000) and stirred to estimate the emulsification tendency. Finally, the % transmittance of the obtained dispersion was measured using a UV-visible spectrophotometer UV-1700 (Shimadzu, Kyoto, Japan) set at 638 nm.

### 2.5. Preparation of SNEDDS Formulation

Various surfactants (Tween-20, Tween-80, Labrasol, Kolliphor-EL, and HCO-30) were used to prepare the SNEDDS formulations. The prepared formulations consisted of a surfactant, propylene glycol (cosurfactant), and Imwitor 988 (oil) in a ratio of 7:1:2. This ratio was selected based on preliminary experiments to optimize the drug loading. The oil content was minimized owing to low febuxostat solubility, whereas the surfactant content was maximized owing to enhanced drug solubility. Propylene glycol was reduced owing to limited drug solubilization capacity, and 10% *w*/*w* was sufficient for SNEDDS solidification. Accurately weighed components were placed in a 5 mL glass beaker and stirred to ensure the formation of a homogenous mixture. For thermoresponsive SNEDDS, poloxamer was added to the SNEDDS formulation and placed in an incubator set at 40 °C until the polymer dissolved.

### 2.6. Design of Experiments

The impact of two independent factors (namely, polymer concentration and molecular weight) on the melting responses of SNEDDS formulations was investigated using Design-Expert software (version 13, Stat-Ease Inc., Minneapolis, MN, USA). To achieve this, a response surface methodology, specifically an I-optimal design, was employed by varying polymer concentrations (2–10% *w*/*w*) and molecular weights (both low and high). Poloxamer 188 (~8400 Da) had a low molecular weight, whereas poloxamer 407 (~12,600 Da) had a high molecular weight. Table 1 presents the 15 formulations suggested by the Design-Expert software. The suggested formulations were prepared to investigate the impact of the selected factors on melting point and melting time. All formulations contained the specified percentage of the tested polymer, 10% *w*/*w* propylene glycol, and a mixture of Tween 20 and Imwitor 988 (in a ratio of 7:2). The formulations (1 g) were placed in test tubes and left to solidify in the refrigerator, following which the melting point and melting time were measured. Statistical analysis was performed using ANOVA to determine the significance of the model terms and their interactions.

### 2.7. Preparation of Capsule-Filled Formulation

The optimized SNEDDS formulation, which was suggested by Design-Expert software based on desirable responses, was prepared, and febuxostat was added at a loading capacity of 40 mg/g. This optimized SNEDDS formulation was immediately added to hard gelatin capsules using a pipette. Subsequently, the formulation was left to solidify in the refrigerator before pharmaceutical assessment.

### 2.8. Melting Point Measurement

The melting point of T-SNEDDS formulations was determined using visual assessment, following the approach established by Kosaraju et al., with appropriate modifications for SNEDDS systems [20]. A controlled water bath system was used to determine the temperature at which complete solid-to-liquid phase transition occurs. A water bath set at 28 ± 0.5 °C was used to measure the melting point. The formulations were placed in glass test tubes for equilibration in a water bath before experimentation. The melting of the formulation was visually inspected before the water bath temperature was increased. The temperature was raised (0.5 ± 0.1 °C) and kept for equilibration before inspection for melting. These steps were repeated until the melting points of all formulations were determined.

### 2.9. Melting Time Measurement

The temperature for the water bath was set at 40 ± 0.1 °C. The melting time was determined separately for each formulation. The solidified formulation was placed in a water bath, and the time required for its conversion to the liquid state following immersion was estimated using a stopwatch.

### 2.10. In-Vitro Melting Assessment

This test was performed on the optimized SNEDDS to assess the in-vivo melting encountered after oral administration. A cylindrical beaker containing approximately 250 mL of water was preheated to 37 °C at the beginning of the experiment to simulate body temperature. The optimized SNEDDS was filled with hard gelatin capsules supplied by Capsugel (Morristown, NJ, USA). The filled capsule was carefully placed within a cylindrical wire to prevent floating and ensure complete immersion in the test medium. The physical appearance of the formulation during the melting process was visually and photographically documented, showing a phase transition from the solid to the liquid state.

### 2.11. Dissolution Study

The dissolution performance of the febuxostat was evaluated using a paddle-type dissolution apparatus (LOGAN Inst. Corp., Somerset, NJ, USA). The release profiles of the following were evaluated and compared: (1) unprocessed febuxostat drug substance; (2) commercially available marketed tablets (clinical reference); and (3) the optimized SNEDDS formulation (developed during this study). Raw febuxostat (40 mg) and equivalent doses of the optimized SNEDDS formulation were accurately weighed into hard gelatin capsules. The dissolution medium, comprising 900 mL of phosphate buffer (pH 6.8), was used to simulate the small intestinal environment in which primary drug absorption occurs. The dissolution medium was preheated to 37 ± 0.5 °C before starting the experiment. During the experiments, the paddle speed was set to 50 rpm. The samples were withdrawn at predetermined intervals using a syringe connected to a filter.

## 3. Results and Discussion

### 3.1. Selection of Oil

**Figure 1:**
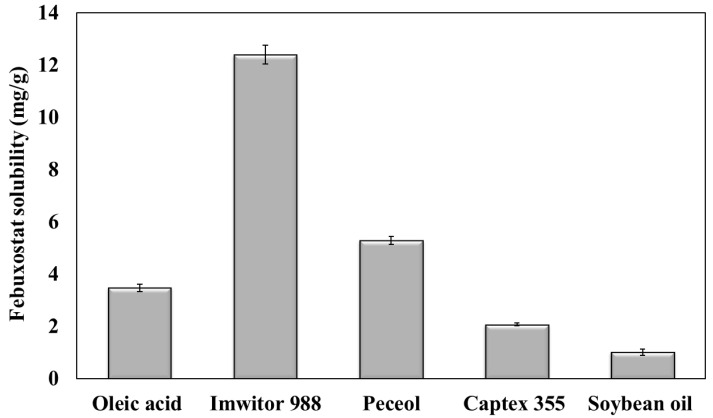
Febuxostat solubility in different oils.

Figure 1 presents the solubility of febuxostat in different types of oils. Febuxostat exhibited the lowest solubility in Captex 355 and soybean oil, which are composed of triglycerides, with a solubility of 2.08 ± 0.05 and 1.02 ± 0.13 mg/g, respectively. Furthermore, its solubility in oleic acid, which is composed of free fatty acids, increased to 3.48 ± 0.13 mg/g. In peceol and Imwitor 988, which are composed of monoglycerides, the solubility of febuxostat was 5.31 ± 0.15 and 12.41 ± 0.36 mg/g, respectively.

The higher solubility of febuxostat in oleic acid than in Captex 355 and soybean oil could be attributed to the hydrogen bond between the drug and the free carboxylic groups of oleic acid [21]. However, esterification of fatty acids with glycerol in triglycerides (Captex 355 and soybean oil) hinders hydrogen bond formation with the drug. Oils containing high amounts of monoglycerides (namely, peceol, and Imwitor 988) exhibited increased febuxostat solubility. This can be attributed to the formation of hydrogen bonds between the hydroxyl groups present in the glycerol [22]. Moreover, monoglycerides can increase the solubility of febuxostat because of their emulsification properties [23].

Imwitor 988 oil was selected to formulate T-SNEDDS in this study due to its high drug solubility. This provides a substantial advantage in incorporating the required dose into a small volume of formulation, thereby reducing the overall dosage volume [24]. Moreover, selecting oils with high drug solubility ensures drug solubilization following oral administration into the lumen of the gastrointestinal tract [25]. This boosts drug bioavailability through the expected concentration gradient [26].

### 3.2. Selection of Surfactant

**Figure 2:**
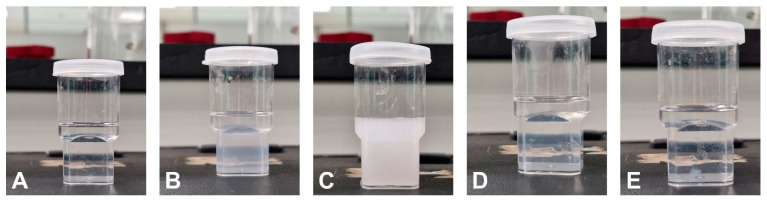
Physical appearance of the aqueous dispersion SNEDDS formulations containing (**A**) Tween-20, (**B**) Tween-80, (**C**) Labrasol, (**D**) Kolliphor EL, and (**E**) HCO-30 surfactants.

The emulsification capacities of Tween-20, Tween-80, Labrasol, Kolliphor EL, and HCO-30 were mixed with a cosurfactant (propylene glycol) and optimum oil (Imwitor 988) to select the surfactant for SNEDDS preparation. The dispersed mixtures (surfactant + cosurfactant + Imwitor 988) in the aqueous phase are shown in Figure 2. The transmittance percentage was assessed using a UV spectrophotometer, and the obtained data are presented in Table 2. The SNEDDS formulation containing labrasol did not form a clear emulsion system, indicated by a low transmittance value (<1%). This can be ascribed to the presence of triglycerides, which hindered the formation of small nanoparticles. However, Tween 80 formed smaller particles, which could be ascribed to the higher emulsification efficiency of the Tween surfactants. However, the presence of oleic acid (a long-chain fatty acid) within its structure hinders efficient packing at the oil–water interface. The other surfactants (tween-20, Kolliphor-EL, and HCO-30) successfully formed nanoemulsion systems, indicated by their high transmittance value (>90%) [27].

Drug solubility in the SNEDDS formulations was measured to determine the optimum surfactant component (Table 2). The SNEDDS formulation comprising Tween-20 exhibited high solubilization power for febuxostat (39.45 ± 0.36 mg/g) compared to other formulations prepared using different surfactants. Thus, Tween-20 was chosen as the surfactant owing to its higher emulsification capacity for Imwitor 988 and superior solubilization power.

### 3.3. Investigating the Independent Factors’ Influence

Table 3 summarizes the melting point and melting time values for the freshly prepared formulations.

**Figure 3:**
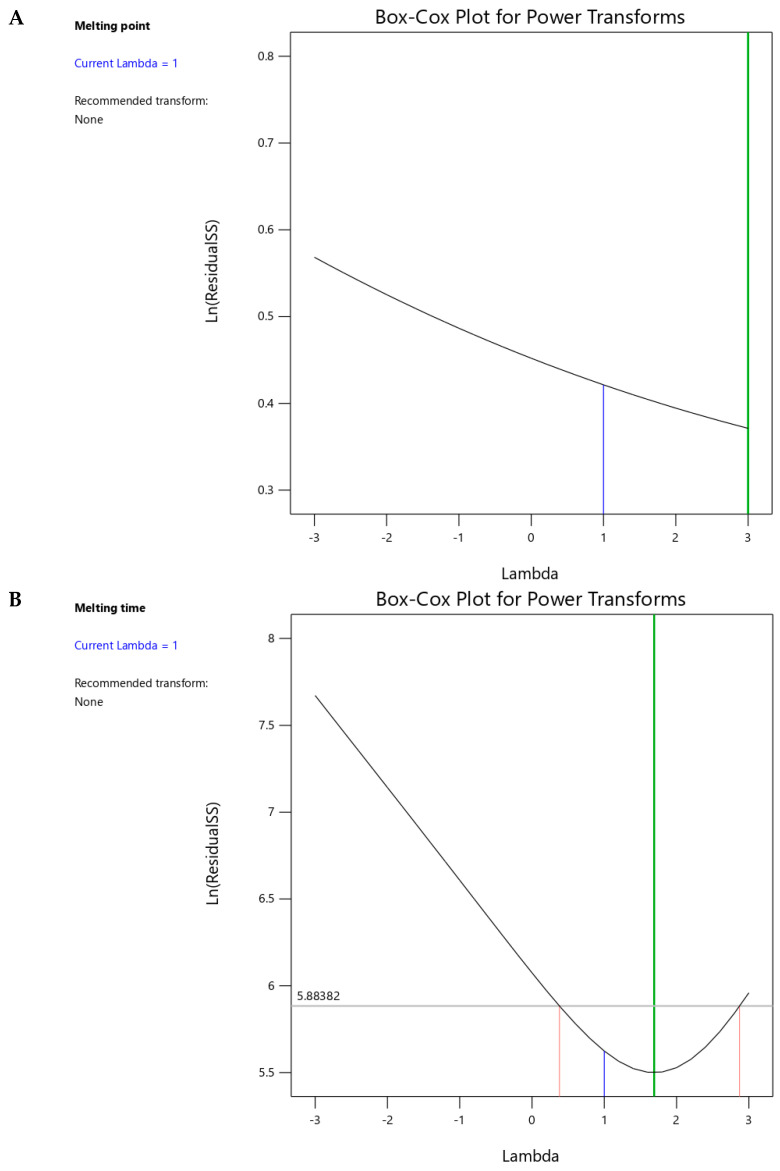
Box–Cox plots for power transformations of (**A**) melting point and (**B**) melting time responses.

ANOVA was performed using DoE to statistically evaluate the influence of independent factors (polymer concentration and molecular weight) on the melting point and time. Based on statistical significance, quadratic and 2FI models were selected for the melting point and time, with F-values of 37.58 (*p* < 0.0001) and 110.34 (*p* < 0.0001), respectively (Table 4). The selected models for measuring melting point and time demonstrated excellent fit, as indicated by the calculated R^2^ values of 0.9376 and 0.9707, and a non-significant lack of fit with p-values of 0.0699 and 0.1029, respectively. The calculated adequate precision ratios were 18.295 and 29.2014, indicating acceptable signal-to-noise characteristics for both models. Box–Cox plots for the melting point and time are presented in Figure 3A,B. The estimated lambda (λ) value for both responses was one, and the recommendation to avoid transformation indicates the suitability of the selected models.

### 3.4. Melting Point

**Figure 4:**
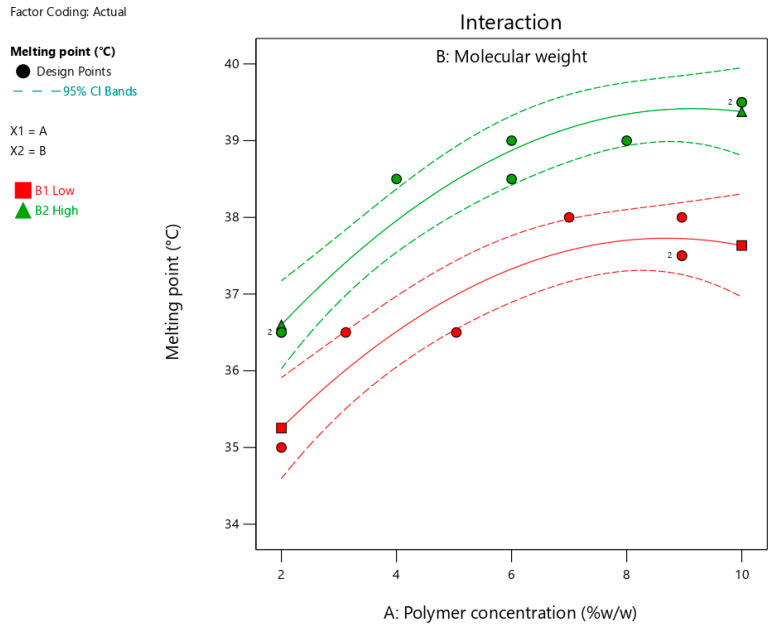
Interaction plot showing the impact of polymer concentration on the melting point at different molecular weights. Red and green lines represent polymers with low and high molecular weights, respectively.

Table 3 summarizes the melting points for the suggested 15 formulations, which ranged from 35 to 39.5 °C. Figure 4 shows the interaction plots for the impact of polymer concentration and molecular weight on the melting points. Table 5 summarizes the statistical analysis of the effect of polymer concentration and molecular weight on the measured melting point and time using Design-Expert software. As shown in Figure 4 and Table 5, increasing the polymer concentration and using a polymer with high molecular weight significantly (*p* < 0.0001) increased the melting point. In addition, the significant (*p* = 0.0074) effect of the quadratic term (polymer concentration^2^) indicated a nonlinear relationship between the polymer concentration and melting point. The influence of the studied factors (polymer concentration and molecular weight) on the melting point can be estimated from the coded equation (Equation (1)). The high coefficient for the polymer concentration (1.29) indicates its predominant influence on the melting point compared with the low value for the polymer molecular weight (0.77).

Y_1_ = 38.10 + 1.29 X_1_ + 0.77 X_2_ + 0.10 X_1_ × X_2_ − 0.88 X_1_^2^ (1)

where Y_1_ = melting point (°C), X_1_ = polymer concentration and X_2_ = molecular weight.

Increasing the concentration of polymers (poloxamers 188 and 407) likely formed a rigid crosslinked matrix, which required high energy to break the bonds and melt the formulation. This finding is in agreement with a previously published study that revealed the positive impact of polymer concentration on intermolecular bonding [28]. Furthermore, increasing the molecular weight of the polymer by increasing the chain length significantly increased crosslinking within the matrix of the formulation. These findings align with those of a previously published study, which reported an increase in intermolecular interactions with an increase in polymer chain length [29]. Therefore, a higher temperature is required to melt a formulation containing long-chain polymers than their counterparts with short chain lengths.

### 3.5. Melting Time

**Figure 5:**
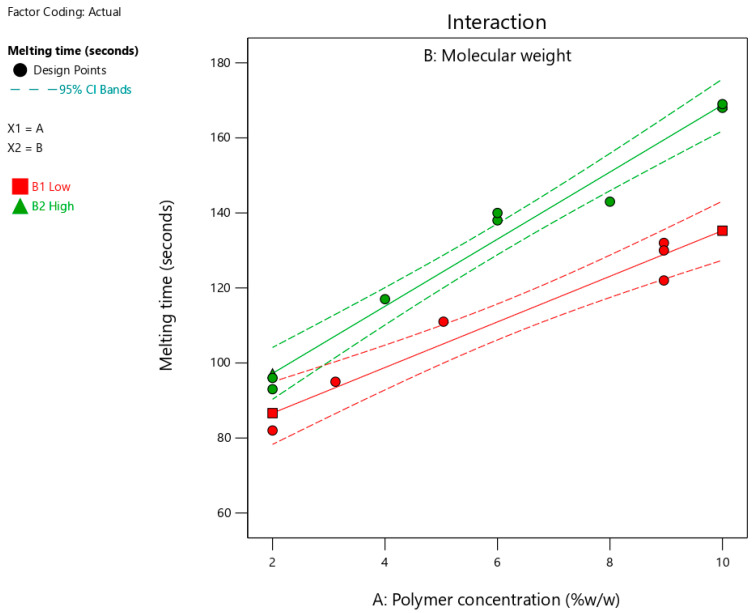
Effect of polymer concentration on melting time at different molecular weights. Red and green lines represent polymers with low and high molecular weights, respectively.

Table 3 summarizes the determined melting times for the suggested fifteen formulations, which ranged from 82 to 169 s. Figure 5 shows the interaction plots for the impact of polymer concentration and molecular weight on the melting time. As shown in Figure 5 and Table 5, increasing the polymer concentration and using a polymer with high molecular weight significantly (*p* < 0.0001) increased the melting time. The significant (*p* = 0.0138) interaction between the two factors indicated that the effect of polymer concentration on the melting time depended on the molecular weight. The influence of the studied factors (polymer concentration and molecular weight) on the melting time can be estimated from the coded equation (Equation (2)). The high coefficient for polymer concentration (30.05) indicates its predominant influence on melting time compared to the low value for polymer molecular weight (11.03).

Y_2_ = 121.97 + 30.05 X_1_+ 11.03 X_2_ + 5.73 X_1_ × X_2_ (2)

where, Y_2_ is the melting time (seconds), X_1_ = polymer concentration, and X_2_ = molecular weight.

The longer time required for complete melting of the formulation with increasing polymer concentration could be due to the presence of a high degree of crosslinking within the solid formulation [30]. Consequently, it took longer to disrupt the intermolecular bonds and facilitate the melting of the formulation. The positive correlation between polymer molecular weight and melting time may result from a high degree of crosslinking within the matrix of the formulation. Thus, the melting time for formulations containing long-chain polymers is longer than their counterparts with short chain lengths. Moreover, the observed interaction between the two factors could be attributed to the entanglement of the polymer chains. Therefore, a formulation containing a higher polymer concentration requires a longer disentanglement time, particularly when a polymer with high molecular weight is used. This indicates a synergistic effect between the two factors, which could be due to the presence of the intermolecular forces that must be overcome during melting.

### 3.6. Optimization Process

A formulation that melts close to 36.5 °C ensures the in-vivo dispersion of nanoemulsion and evades premature in-vitro melting with an extraordinary safety margin. Minimizing the melting time ensures rapid melting after oral administration and subsequent exposure to body temperature.

**Figure 6:**
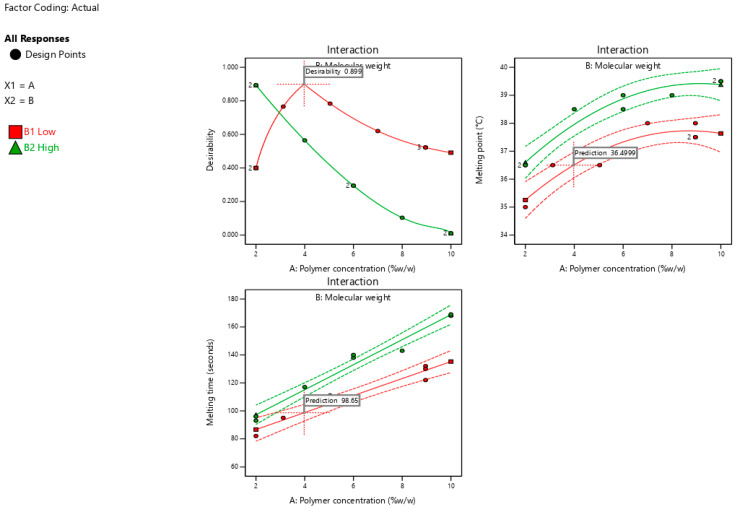
Optimization plots show the suggested formulation’s desirability (0.899) along with prediction values for melting temperature (36.5 °C) and melting time (98.65 s) based on polymer concentration and molecular weight interactions.

Figure 6 shows the optimization plots for desirability, highlighting the optimal regions where the interactions between polymer concentration and molecular weight meet the selected criteria. The suggested formulation consisted of a low-molecular-weight polymer (poloxamer 188) at a concentration of 3.98% *w*/*w*. A dashed-plus sign with a desirability value of 0.899 represents the suggested formulation. The expected responses, including the melting point and melting time, for the optimized SNEDDS formulation were 36.5 °C and 98.65 s, respectively.

### 3.7. Validation of Design of Experiments

Table 6 presents the predicted and mean values of the melting point and time. The optimum formulation exhibited a melting point of 36 °C and a melting time of 111.33 s. These results are close to the predicted mean values, indicating the reliability of the designed model for predicting the response values.

### 3.8. In-Vitro Melting Assessment

**Figure 7:**
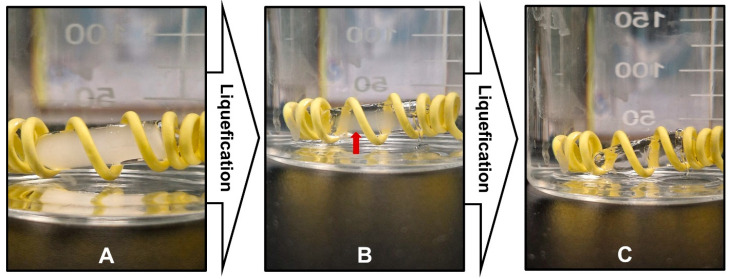
In-vitro melting behavior of optimized SNEDDS formulation showing phase transition from solid to liquid state when exposed to aqueous medium at 37 °C. (**A**) Initial solid state of SNEDDS formulation. (**B**) Intermediate melting stage with gradual phase transition (indicated by red arrow). (**C**) Complete liquid state after melting.

Figure 7 shows that the optimized SNEDDS formulation successfully melted after exposure to body temperature. The encapsulated formulation transformed from a solid to a liquid state at physiological body temperature. Figure 7A shows the solid formulation at the beginning of the experiment, whereas Figure 7B shows that the formulation started to undergo gradual melting, as indicated by the red arrow. Finally, Figure 7C shows that the formulation was completely melted without any signs of drug precipitation or phase separation in the SNEDDS components.

The melting behavior of the SNEDDS formulation can be attributed to the unique thermal behavior of poloxamer 188 incorporated into the formulation [31]. Polymer units are discretely present and form hydrogen bonds with the propylene glycol molecules [14,15]. The crosslinked networks formed within the SNEDDS formulation triggered their solidification. Moreover, the former network is further stabilized by reported interactions (hydrophobic and van der Waals forces) between the poloxamer molecules through polypropylene oxide blocks [32]. Exposure to temperatures above the critical micellization temperature disrupts the hydrophobic interactions and van der Waals bonds, which trigger the arrangement of polymers in a spherical micellar structure [33]. This break formed crosslinked networks and allowed the solubilization of the polymeric micellar structure within the formulation.

### 3.9. Dissolution Study

**Figure 8:**
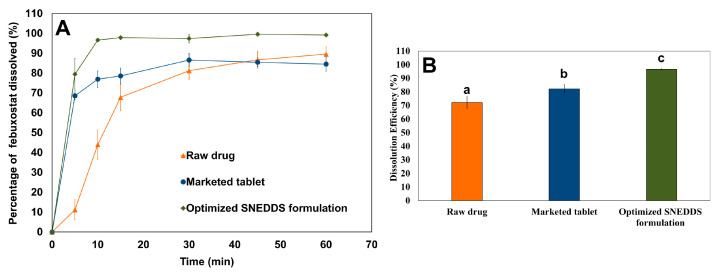
Comparative in-vitro dissolution profiles showing (**A**) dissolution profile and (**B**) dissolution efficiency for raw febuxostat, marketed tablet, and optimized SNEDDS formulation in phosphate buffer (pH 6.8) at 37 °C. Different letters indicate statistically significant differences, with a *p*-value < 0.05.

The dissolution profiles of febuxostat obtained using different agents are shown in Figure 8. The optimized SNEDDS formulation rapidly melted and dispersed in the dissolution medium, as indicated by the percentage of drug dissolved (79.5 ± 8.0%) after 5 min. This finding confirms that the solidification technology used in this study does not substantially influence the emulsification behavior of the SNEDDS components. Furthermore, the optimized SNEDDS formulation enhanced the dissolution rate of febuxostat compared with that of the raw material. This can be attributed to the amorphization and solubilization of the dispersed formulation components in the medium. Additionally, the optimized SNEDDS formulation (96.66 ± 0.28%) significantly (*p* < 0.05) enhanced the dissolution efficiency of febuxostat compared with the raw drug (72.09 ± 4.33%). These findings align with those of previous studies, which demonstrated that SNEDDS formulations significantly enhance the dissolution rate and extent of poorly water-soluble drugs [5,34].

The dissolution efficiency for the marketed tablet was calculated to be 82.23 ± 3.10%. However, the optimized SNEDDS formulation demonstrated superior dissolution performance, with a statistically significant enhancement (*p* < 0.05) in dissolution efficiency. This could be attributed to the dispersion of the drug in an amorphous state within the optimized SNEDDS formulation, which makes it available for dissolution [35]. Moreover, emulsification of SNEDDS into nanoemulsions provides favorable conditions for drug solubilization [36]. These findings revealed that the approach used showed promise in solidifying SNEDDS and preventing formulation leakage compared to available alternative methods.

## 4. Conclusions

In this study, an optimized SNEDDS formulation loaded with febuxostat was prepared using a DoE approach. The developed SNEDDS formulation demonstrated an outstanding temperature-dependent phase transition behavior. It remained solid at room temperature and rapidly melted under physiological conditions when exposed to body temperature. The optimized SNEDDS formulation exhibited significantly enhanced dissolution performance compared to both the raw drug and marketed tablet formulations. This promising technology represents a simple and practical advancement in the development of solid SNEDDS formulation.

## Figures and Tables

**Table 1:** Suggested fifteen formulations.

Run	Polymer Concentration (% *w*/*w*)	Molecular Weight
1	2	High
2	10	High
3	8.96	Low
4	8	High
5	2	High
6	5.04	Low
7	6	High
8	8.96	Low
9	7	Low
10	6	High
11	10	High
12	4	High
13	3.12	Low
14	2	Low
15	8.96	Low

**Table 2:** Physical appearance of dispersed SNEDDS, their measured transmittance, and drug solubility.

Surfactant Type in SNEDDS Formulation	Physical Appearance	Transmittance (%)	Solubility (mg/g)
Tween-20	Transparent	97.73 ± 0.21	39.45 ± 0.36 ^a^
Tween-80	Whitish appearance	70.73 ± 0.06	38.68 ± 0.25 ^a^
Labrasol	Turbid	0.30 ± 0.00	36.19 ± 0.65 ^b^
Kolliphor-EL	Slightly bluish	92.03 ± 0.15	36.86 ± 0.46 ^b^
HCO-30	Transparent	96.13 ± 0.23	32.05 ± 0.61 ^c^

Different letters indicate significance; *p*-value 0.05.

**Table 3:** The measured melting point and melting time for the fifteen formulations.

Runs	Melting Point (°C)	Melting Time (s)
1	36.5	93
2	39.5	168
3	37.5	132
4	39	143
5	36.5	96
6	36.5	111
7	39	140
8	37.5	122
9	38	141
10	38.5	138
11	39.5	169
12	38.5	117
13	36.5	95
14	35	82
15	38	130

**Table 4:** ANOVA analysis of the measured responses for the selected models.

Response	Selected Model	Freedom Degree	*p*-Value	Lack of Fit *p*-Value	R^2^	Adequate Precision
Melting point (°C)	Quadratic	4	<0.0001	0.0699	0.9376	18.2950
Melting time (seconds)	2FI	3	<0.0001	0.1029	0.9707	29.2014

**Table 5:** ANOVA summary showing the estimated *p*-values for the effects of independent variables on the measured responses.

Statistical Parameters	Melting Point	Melting Time
Polymer concentration	<0.0001	<0.0001
Molecular weight	<0.0001	<0.0001
Two-factor interaction	0.5031	0.0138
polymer concentration^2^	0.0074	---

**Table 6:** Predicted and data mean values for measured responses of the prepared optimized SNEDDS formulation.

Measured Response	Predicted Mean	Data Mean
Melting point (°C)	36.5	36
Melting time (seconds)	98.65	111.33

## Data Availability

Data are available in the manuscript.
